# Expressions of matrix metalloproteinase-9 (MMP-9), dentin sialophosphoprotein (DSPP), and osteopontin (OPN) at histologically negative surgical margins may predict recurrence of oral squamous cell carcinoma

**DOI:** 10.18632/oncotarget.373

**Published:** 2012-03-11

**Authors:** Kalu U.E. Ogbureke, Paul M. Weinberger, Stephen W. Looney, Li Li, Larry W. Fisher

**Affiliations:** ^1^ College of Dental Medicine, Georgia Health Sciences University, 1120 15th Street, Augusta, GA; ^2^ Medical College of Georgia, Georgia Health Sciences University, 1120 15th Street, Augusta, GA; ^3^ College of Graduate Studies, Georgia Health Sciences University, 1120 15th Street, Augusta, GA; ^4^ Craniofacial and Skeletal Disease Branch, National Institute of Dental and Craniofacial Research, NIH, 30 Convent Drive, Building 30, Room 228, Bethesda, MD

**Keywords:** Oral Cancer, DSPP, BSP, OPN, MMPs, Tumor-Free Margin

## Abstract

Up to 50% of oral squamous cell carcinomas (OSCCs) recur following surgical resections with conventional “histologically-negative” margins. Three members of the SIBLING (Small Integrin Binding LIgand N-linked Gylcoprotein) family of proteins: dentin sialophophoprotein (DSPP); bone sialoprotein (BSP); and osteopontin OPN are upregulated in OSCCs. In this study, we aimed to correlate the expression of DSPP, OPN and BSP as well as three SIBLING-partners, matrix metalloproteinase-2 (MMP-2), matrix metalloproteinase-3 (MMP-3), and matrix metalloproteinase-9 (MMP-9), at histologically-negative margins of OSCCs with tumor recurrence. Immunohistochemical analyses of the SIBLINGs and MMP expressions at histologically-negative margins of OSCC was carried out in a retrospective study of 20 patients, and the results correlated with tumor recurrence. Each protein was dichotomized as “present” (≥10% staining) or “absent” (< 10% staining). The Sensitivity, Specificity, Positive Predictive Value(PV+) and Negative Predictive Value (PV−) for recurrence was calculated for each protein, along with their overall diagnostic accuracy, calculated as: (number of true positives + number of true negatives)/ number of patients. OSCC recurred in 9 of 20 patients (45%), a ratio not significantly different from the estimated population recurrence rate of 50% (p = 0.664). Among the SIBLINGs, DSPP and OPN showed the greatest Accuracy with DSPP being more Sensitive (89%) and OPN more Specific (64%). MMP-9 showed the greatest overall Accuracy (80%), slightly less Sensitivity (67%) and more Specificity (100%), than either DSPP or OPN. MMP-9 showed a superior positive PV than either DSPP or OPN. The negative PVs of OPN and MMP-9 were almost identical, and inferior to DSPP. We conclude that DSPP, OPN, or MMP-9 expressions at histologically-negative surgical margins predict OSCC recurrence with MMP-9 being the preferred predictor. These proteins may identify patients who could benefit from more extensive resection, or from adjunct treatments such as radiotherapy.

## INTRODUCTION

Most mortality in oral squamous cell carcinoma (OSCC) patients is due to local recurrent disease and regional spread following surgical treatment failure at the primary site [1-5]. In treating primary OSCC, the surgeon aims to achieve total ablation of the tumor because inadequate resection leaves the patient with an increased chance of disease recurrence [1-6]. Surgical excision of OSCC with a curative intent is currently guided mostly by obtaining histologically tumor-free (negative) margins [4-6]. A negative surgical resection margin is defined as a 5-10 mm margin of tissue beyond the edge of the tumor that histologically lacks evidence of invasive carcinoma, carcinoma-in-situ, or any degree of dysplasia [2]. The histologic status of a resection margin has long been used as a potential indicator for recurrence and prognosis, and also is used to make decisions regarding the need for adjuvant radiation therapy [2-7].

However, up to 50% of OSCCs recur following surgical intervention even with “adequate tumor-free” (histologically-negative) margins, usually within 2 years of initial surgical intervention [3, 5]. This high recurrence rate at primary tumor sites suggests malignant transformation at the molecular level that may precede the phenotypic histologic changes observed. Therefore, the practical aspects of histologically defined negative margins are inadequate in determining recurrence-free status following surgical treatment in OSCC patients [6]. Recent studies explored the utility of molecular markers, independently or as complementary to the histologic parameters, to define functionally better resection margins that result in recurrence-free status (RFS) for patients treated for primary OSCCs as well as other head and neck cancers [4-9]. However, most markers reported to date lack the sensitivity and/or ease of applicability required for routine clinical use [4-6].

Dentin sialophosphoprotein (DSPP), bone sialoprotein (BSP), and osteopontin (OPN) are three members of the Small Integrin-Binding LIgand N-linked Glycoprotein (SIBLING) family of proteins [10] reported to be up-regulated in a number of cancers, including breast, lung, prostate, and OSCCs [11, 12]. The other two members of the SIBLING family are dentin matrix protein 1 (DMP1), and matrix extracellar phosphoglycoprotein (MEPE) [10]. Because BSP, DSPP, and OPN were upregulated in OSCCs, while DMP1 and MEPE were absent, we designated BSP, DSPP, and OPN as oral cancer-associated SIBLNGs. Furthermore, DSPP expression was associated with histological markers of aggressiveness of OSCCs [12], and its expression in resected dysplastic oral premalignant lesion (OPLs) was correlated with subsequent occurence of invasive OSCC [13].

Three members of the SIBLING gene family also have been determined to specifically bind and activate three different matrix metalloproteinases (MMPs): BSP with MMP-2; OPN with MMP-3; and DMP-1 with MMP-9 [14]. The binding of SIBLING to their corresponding proMMPs results not only in making the proMMPs enzymatically active, but also in reactivating the TIMP (tissue inhibitors of MMP) inhibited MMPs [14]. The pro and active MMP-SIBLING complexes are disrupted by serum complement Factor H, thereby providing a rate-limiting step in the SIBLING-MMP interaction as well as confining activity to the vicinity of secretion in vivo [14]. The SIBLING-MMP interaction offers an insight into alternative methods of regulating the activity of at least three MMPs. The SIBLING-MMP co-localization has also been shown to exist in vivo [12, 15, 16]. The cognate MMPs for DSPP and MEPE, if any, are yet to be determined.

In the present retrospective study, we investigate the expression, at histologically-negative resection margins of primary OSCCs, all of the three oral cancer-associated SIBLINGs (BSP, OPN, DSPP) as well as three MMPs (MMP-2, MMP-3, and MMP-9) and correlate expression with the clinical outcome parameters, recurrence and survival.

**Figure 1:**
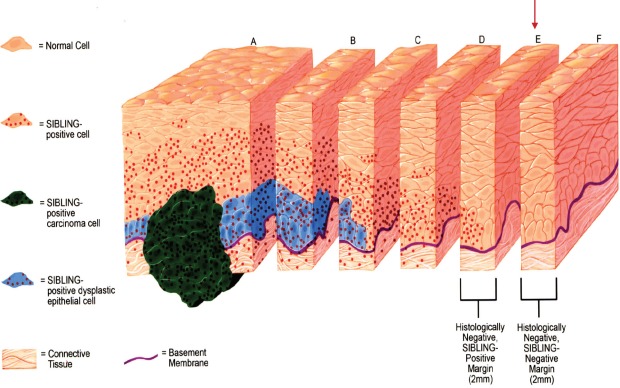
Schematics illustration of Tumor Margin Estimation with SIBLINGs and MMPs at resection margins, and the process of determining SIBLING/MMP-positive margins via level and serial sectioning at histologically negative resection margins of OSCC Slice “A” represents histologically positive-SIBLING positive resection margin for invasive OSCC (green) and dysplastic epithelium (blue), while slices “B” and “C” illustrate histologically positive-SIBLING positive resection margins for dysplastic epithelium (blue). Slices “D” and “E” illustrates histologically negative resection margins, while Slice “F” illustrates histologically negative-SIBLING negative resection margin. SIBLING positivity at margins is indicated by red dots (absent in slice F).

**Figure 2:**
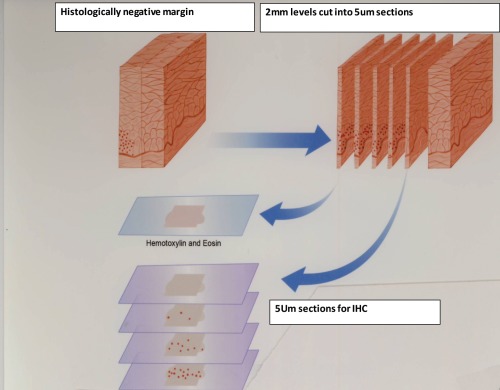
Schematics of steps for preparation of 5μm slides for the immunohistochemistry investigation of SIBLINGs/MMPs expression at histologically negative margins (Slices D, E, F) from Figure 1 as described

## RESULTS

### SIBLING and MMP expression at histologically negative resection margins

**Figure 3:**
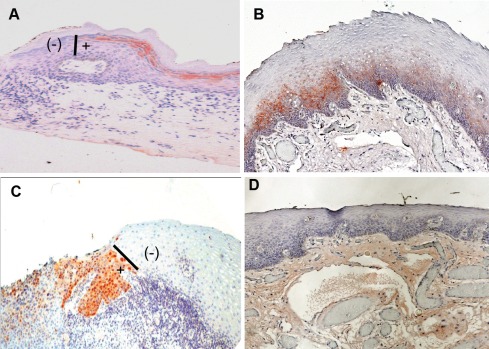
SIBLING/MMP immunoreactivity at histologically negative resection margins of OSCC (**A**) Example of DSPP expression scored as “1+” indicating >10% <50% of cells at resection margin showing immunoreactivity with DSPP (monoclonal antibody, LF-Mb21). (**B**) Expression scored as “2+” where 50% <75% of cells at histologically negative margins show immunoreactivity to BSP (antibody, LF-Mb25). (**C**) Expression scored as “3+” with more than 75% of normal epithelial cells at resection margins positive with MMP-9 (antibody LF-184). (D) Representative non-immune control showed expression scored as 0 for no immunoreactivity at histologically negative margin. Chromogenic staining (reddish-brown color) was achieved with 3-amino-9-ethylcarbazole (AEC) and counterstained with hematoxylin. Bars (**I**) in **A** and **C** show point of abrupt end of DSPP and MMP-9 expression, respectively, at the margins demarcating area of histologically negative but DSPP/MMP-9 positive (**+**) from histologically negative and DSPP/MMP-9 negative (**-**) portions of the margin.

A total of 200 histologically negative surgical mucosal margin sections (average of 10 sections per case), obtained at several consecutive levels, from the 20 cases of OSCCs were each subjected to immunohistochemistry analysis for the expressions of each of the SIBLINGs (BSP, DSPP, OPN), and MMPs (MMP-2, MMP-3, MMP-9). As shown in Table 2, nine (45%) of the cases showed positivity for at least one SIBLING or MMP on at least one histologically negative resection margin. Immunoreactivity for the SIBLINGs and MMPs exhibited similar pattern of expression, and representative results are shown in Figure 3 with 3A representing a semiquantitative score of “1+” (DSPP), 3B a score of “2+” (BSP), and 3C a score of “3+” (MMP-9). Figure 3D is a representative negative control (non-immune serum). Chromogenic staining (reddish-brown color) was achieved with 3-amino-9-ethylcarbazole (AEC) and counterstained with hematoxylin. The vertical bars (**I**) shown in Figures 3A and 3C indicate point of abrupt disruption of DSPP and MMP-9 expression, respectively, at margin demarcating area of histologically negative but DSPP/MMP-9 positive (**+**) from histologically negative and DSPP/MMP-9 negative (**-**) portions of the margins. This demarcation also provided an in-section negative control.

**Table 1:** Clinical characteristics and variables of tumors related to tumor resection margins

Serial #	Age	Gender	Ethnicity	Subsite	T	N	M	TNM (Stage)	Diff	Invasion	PN Spread	Radiotherapy (RT)
1	58	M	AA	TG	2	0	0	2	2	0	1	0
2	63	M	C	FOM	2	0	0	2	3	1	1	3
3	44	M	C	TG	4	2	0	4	1	1	1	3
4	54	F	AA	TG	2	0	0	2	2	0	0	3
5	51	M	C	TG	X	X	X	X	3	1	1	1
6	33	M	C	TG	2	0	0	2	2	0	0	0
7	50	M	C	FOM	2	0	0	2	2	0	0	4
8	53	M	AA	SP	1	0	0	1	3	0	0	0
9	45	F	C	TG	2	1	0	3	2	0	0	3
10	48	M	C	FOM	2	0	0	2	2	0	0	0
11	52	F	Other	BM	4	2a	0	4	3	1	0	3
12	85	F	C	FOM	1	0	0	1	3	0	0	0
13	68	F	C	BM	3	0	0	3	3	0	0	3
14	68	F	C	TG	2	2a	0	4	3	1	1	3
15	51	M	C	TG	4	2a	0	4	3	1	0	3
16	53	F	AA	RMT	4	1	0	4	2	1	X	3
17	58	F	C	BM	4	0	0	4	3	0	1	3
18	49	M	C	RMT	2	0	0	2	3	X	X	0
19	54	M	C	FOM	4	2	0	4	3	0	0	3
20	61	M	C	FOM	4	2b	0	4	3	1	1	3

Keys:

**Diff** = Differentiation: 1=poorly diff; 2=moderately diff; 3= well diff

**LV** = presence or absence of lymphatic/vascular invasion: as binary logicals (Yes =1; No=0)

**PN** spread= Presence/Absence of perineural spread: as binary logicals (Yes=1; No=0)

Radiotherapy (**RT**): 0= no RT; 1= primary RT; 2= chemo/RT; 3= post-op RT (administered after biopsy specimen obtained); 4= re-irradiation for recurrence Stage”

**X** = not detectable

**T** = Tumor Size; **N**= regional node status; **M**= presence/absence of distant metastasis

Subsite: **TG**= tongue; **BM**= buccal mucosa; **RMT**= retromolar trigone; **FOM**= floor of mouth; **SP**= soft palate

**Table 2:** Semiquantitative scores for SIBLING and MMP immunoreactivity at tumor resection margins

Serial #	Primary Tumor (PT)							Adjacent Mucosa/Resection Margin						*Recurrence (PT)
H&E	BSP	DSPP	OPN	MMP2	MMP3	MMP9	BSP	DSPP	OPN	MMP2	MMP3	MMP9
1	Y	3	3	1	0	2	0	2	0	1	1	2	0	N
2	Y	3	2	0	0	2	3	0	0	0	0	0	0	N
3	Y	2	2	0	0	2	2	2	2	0	0	0	0	Y 17mo
4	Y	2	1	0	1	1	0	0	0	0	0	1	0	N
5	Y	3	2	2	2	2	1	2	2	2	1	1	0	Y 24mo
6	Y	2	3	2	1	2	0	2	2	2	1	2	0	N
7	Y	1	0	0	1	1	0	1	0	0	1	0	0	N
8	Y	0	2	0	0	0	1	0	2	0	0	0	0	N
9	Y	1	2	2	1	1	1	0	3	0	2	0	0	N
10	Y	0	3	2	0	0	2	1	2	2	1	0	?	N
11	Y	3	2	0	1	2	3	1	2	1	0	1	1	Y 11.5mo
12	Y	3	3	2	0	3	0	3	2	0	0	3	0	N
13	Y	1	2	2	3	2	2	0	2	1	2	1	2	Y 13mo
14	Y	2	0	2	1	0	0	0	0	0	0	0	0	Y 12mo
15	Y	3	2	1	1	3	3	0	1	2	2	3	3	Y 9mo
16	Y	2	3	3	0	3	3	2	3	3	0	3	3	Y 7mo
17	Y	2	1	1	2	1	0	3	0	0	2	1	0	N
18	Y	2	1	1	2	1	0	2	0	1	2	1	0	N
19	Y	0	2	2	0	3	2	1	3	1	1	2	1	Y 7mo
20	Y	2	2	1	1	2	2	2	2	2	1	1	3	Y 6mo

Key to symbols

Y= Tumor section and margins verified by H&E sections

Y= Recurrent; 9/20 (45%) had clinical documentation of Recurrence

N= absence of recurrence within 3years.

(yr)= interval between initial primary tumor resection and first primary site recurrence

Immunostain scoring is semiquantitative using our previous criteria (recent OPL paper attached).

0= negative immunostain

1=positive cells less than 10%; 2= positive cells more than 50% but less than 75%; 3= diffuse positivity.

* Recurrence-Free Time (RFT) ranged from 6-17months with an Average RFT of 10.5months.

### SIBLING/MMP expression at negative margins versus recurrence

Of the 20 cases in the present study, 9 (45%) had clinical documentation of recurrence of OSCC. This rate does not differ significantly from the estimated population recurrence rate of 50% (p = 0.664). Time between initial surgical resection of primary tumor and initial recurrence (Recurrence-Free Time; RFT) ranged from 6-17 months with an average RFT (ARFT) of 10.5 months (Table 2). When the three SIBLINGs were dichotomized, DSPP and OPN exhibited the greatest overall accuracy (Table 3) with DSPP being slightly more sensitive (89%) and OPN slightly more specific (64%). Similarly, Table 3 shows how dichotomizing the three MMPs showed that MMP-9 had the greatest overall accuracy (80% although slightly less sensitive with a value of 67%) and more specificity (100%) than either DSPP (70% and 55%, respectively) or OPN (70% and 64%, respectively). With respect to positive predictive value (PV+) Table 3 shows that DSPP and OPN were almost identical (62% and 64%, respectively), while MMP-9 (100%) was superior to either. On the other hand, the respective negative predictive values of OPN and MMP-9 were essentially identical and slightly inferior to that of DSPP (Table 3). Overall, MMP-9 appeared to be the preferred predictor of recurrence of OSCC.

**Table 3:** Sensitivity, Specificity, and Predictive Values of Dichotomized SIBLINGs and MMPs as Predictors of Recurrence of OSCC

SIBLING	Sensitivity	Specificity	PV+	PV−	Accuracy
BSP	6/9 (67%)	4/11 (36%)	6/13 (46%)	4/7 (57%)	10/20 (50%)
DSPP	8/9 (89%)	6/11 (55%)	8/13 (62%)	6/7 (86%)	14/20 (70%)
OPN	7/9 (78%)	7/11 (64%)	7/11 (64%)	7/9 (78%)	14/20 (70%)
**MMP**	**Sensitivity**	**Specificity**	**PV+**	**PV−**	**Accuracy**
MMP2	5/9 (56%)	4/11 (36%)	5/12 (42%)	4/8 (50%)	9/20 (45%)
MMP3	7/9 (78%)	5/11 (45%)	7/13 (54%)	5/7 (71%)	12/20 (60%)
MMP9	6/9 (67%)	10/10 (100%)	6/6 (100%)	10/13 (77%)	16/20 (80%)

Based on the Fisher's Exact and Fisher-Freeman-Halton tests (Table 4), tumor size, tumor stage, lymphatic/vascular invasion, node metastasis, and radiotherapy were significantly associated with OSCC recurrence rate whereas mean age did not differ significantly between the recurrence and no-recurrence groups (54 ± 13 vs. 56 ± 8, t = 0.32, d.f. = 18, p-value = 0.752). With the exception of radiotherapy, each of the clinical variables significantly associated with recurrence (Table 4) also were significantly associated with recurrence-free survival, as measured by the unadjusted hazard ratio obtained from the univariate Cox regression analysis (Table 5). Even after dichotomizing radiotherapy into “none vs. any,” the data were insufficient to fit a viable Cox regression model (not shown). Age was not significantly associated with recurrence-free survival (HR 1.01, 95% C.I. 0.95-1.06, p-value = 0.732). Furthermore, as shown in Table 6, the unadjusted hazard ratio from the univariate Cox analysis indicated that DSPP, OPN and MMP-9 were significantly associated with recurrence (*p-values*: DSPP=0.040; OPN=0.050; MMP-9<0.001).

**Table 4:** OSCC Patient and Tumor Characteristics vs. Recurrence

Variable	No. of Patients	No. of Recurrences	P-value
**Race**			
Non-White	5	2	--
White	15	7	1.000
**Gender**			
Female	8	4	--
Male	12	4	0.658
**Subsite**			
BOT	1	1	0.900
Buccal	3	2	
FOM	6	2	
Oral Tongue	7	3	
RMT	2	1	
Soft Palate	1	0	
**Tumor Size**			0.002*
≤ 2 cm	2	0	
> 2 but ≤ 4 cm	9	1	
> 4 cm	1	1	
Tumor Invaded Adjacent Structures	7	6	
**Tumor Stage**			< 0.001*
1	2	0	
2	7	0	
3	2	1	
4	8	7	
**Lymphatic/Vascular Invasion**			
No (Ref)	11	2	--
Yes	8	7	0.006*
**Node Metastasis**			
0	11	1	< .001*
1	2	1	
2	2	2	
2a	3	3	
2b	1	1	
**Differentiation**			
Poor	1	0	0.098
Moderate	6	1	
Good	12	7	
**Peripheral Nerve Spread**			
No	11	4	--
Yes	7	4	0.631
**Radiotherapy**			
No RT	6	0	0.012*
Primary RT	1	1	
Postop XRT	12	8	
Re-irradiation for Recurrence	1	0	

* Statistically significant at p < 0.05

**Table 5:** Significant Unadjusted Hazard Ratios for OSCC Recurrence for Patient and Tumor Characteristics

Variable	No. of Patients	No. of Recurrences	Unadjusted HR (95% C.I.)	P-value
Tumor Size				< 0.001*
≤ 2 cm	2	0	3.76 (1.70-12.15)	
> 2 but ≤ 4 cm	9	1	(variable treated as ordinal)	
> 4 cm	1	1		
Tumor Invades Adjacent Structures	7	6		
Tumor Stage				< 0.001*
1	2	0	7.38 (2.22 -104.99)	
2	7	0	(variable treated as ordinal)	
3	2	1		
4	8	7		
Lymphatic/Vascular Invasion				
No (Ref)	11	2	--	--
Yes	8	7	8.07 (1.90-54.93)	0.004*
Node Metastasis				
No (ref)	11	1	--	--
Yes	8	7	20.01 (3.42-379.91)	< 0.001*

Abbreviation: ref = referent

* Statistically significant at p < 0.05

**Table 6:** Unadjusted Hazard Ratios for OSCC Recurrence for SIBLINGS and MMPs

Variable	No. of Patients	No. of Recurrences	Unadjusted HR (95% C.I.)	P-value
BSP				
No (Ref)	7	3	--	--
Yes	13	6	1.14 (0.30-5.43)	0.849
DSPP				
No (Ref)	7	1	--	--
Yes	13	8	5.90 (1.08-109.63)	0.040*
OPN				
No (Ref)	9	2	--	--
Yes	11	7	4.18 (1.00-28.16)	0.050*
MMP2				
No (Ref)	8	4	--	--
Yes	12	5	0.82 (0.22-3.32)	0.769
MMP3				
No (Ref)	7	2	--	--
Yes	13	7	2.47 (0.59-16.59)	0.227
MMP9				
No (Ref)	13	3	--	--
Yes	6	6	34.55 (5.42-674.15)	< 0.001*

Abbreviation: ref = referent

* Statistically significant at p < 0.05

### SIBLING/MMP expression at negative margins versus clinical variables

Following Fisher's Exact and Fisher-Freeman-Halton test analyses, no patient characteristics or clinical variables were found to be significantly associated with OPN or DSPP status (data not shown). On the other hand, tumor size and node metastasis were significantly associated with MMP-9 expression at negative margins (p-value: Tumor Size=0.002; Node Metastasis=0.043; Table 7). MMP-9 was absent in margins from tumors ≤ 4 cm, but present in 75% of margins from tumors > 4 cm. It was also likely to be present in margin regions from tumors that invaded adjacent structures (T4 tumors; Table 7). MMP-9 was found in only 10% of tumors without later node metastasis, but it was present in 63% of those with subsequent metastases (Table 7). Based on Fisher's Exact results, the presence of DSPP and OPN were significantly associated with the presence of MMP-9 (p-value: DSPP=0.044; OPN=0.011; Table 8). Also, MMP-9 was present at margins that were also positive for DSPP and/or OPN

**Table 7:** OSCC Patient and Tumor Characteristics vs MMP9 Status

Variable	No. of Patients	MMP9 Present	P-value
**Race**			
Non-White	5	2	1.000
White	14	4	
**Gender**			
Female	8	3	1.000
Male	11	3	
**Subsite**			
BOT	1	0	0.610
Buccal	3	2	
FOM	5	2	
Oral Tongue	7	1	
RMT	2	1	
Soft Palate	1	0	
**Tumor Size**			
≤ 2 cm	2	0	0.002*
> 2 but ≤ 4 cm	8	0	
> 4 cm	1	1	
Tumor Invaded Adjacent Structures	7	5	
**Tumor Stage**			
1	2	0	0.051
2	6	0	
3	2	1	
4	8	5	
**Lymphatic/Vascular Invasion**			
No	10	2	0.321
Yes	8	4	
**Node Metastasis**			
No	10	1	0.043*
Yes	8	5	
**Radiotherapy**			
None (ref)	6	0	0.128
Any	14	9	
**Differentiation**			
Poor	1	0	0.726
Moderate	6	1	
Good	12	5	
Peripheral Nerve Spread			
No	6	4	0.338
Yes	6	1	

* Statistically significant at p < 0.05

**Table 8:** SIBLINGS and Other MMPs vs MMP9 Status

Variable	No. of Patients	MMP9 Present	P-value
BSP			
Absent	7	2	1.000
Present	12	4	
DSPP			
Absent	7	0	0.044*
Present	12	6	
OPN			
Absent	9	0	0.011*
Present	10	6	
MMP2			
Absent	8	2	1.000
Present	11	4	
MMP3			
Absent	6	0	0.109
Present	13	6	

* Statistically significant at p < 0.05

### SIBLING/MMP expression at histologically negative margin versus recurrence-free status

When multivariate Cox regression with forward selection was performed for OPN and DSPP with each of the clinical factors that were significantly associated with recurrence-free survival in the univariate Cox analyses, neither SIBLING was retained in the final regression model. That is, SIBLINGs were not independently predictive of recurrence-free survival after accounting for significant clinical factors. However, in the multivariate analysis where MMP-9 was selected for entry into the Cox model [followed by lymphatic/vascular invasion (LVI)], both MMP-9 and LVI were negatively associated with recurrence-free survival after adjusting for the other factor (p-value: LVI=0.006; Table 9). Furthermore, both MMP-9 and tumor size (T) were significantly but negatively associated with recurrence-free survival after adjusting for the other factor in multivariate analysis (p-value: T=0.050; Table 9). The sparseness of the data precluded the possibility of performing a multivariate analysis in order to examine the combination of MMP-9 with the other two clinical factors (tumor stage and nodal status) that were significantly associated with recurrence-free survival in univariate Cox analysis. Multivariate Cox regression with forward selection performed for OPN and DSPP with MMP-9 showed that neither SIBLING was retained in the final regression model; that is, they were not independently predictive of recurrence-free survival after accounting for the effect of MMP-9.

**Table 9:** Adjusted Hazard Ratios for Recurrence by MMP9 Status and Significant Covariates

Covariate	MMP9 Adjusted for Covariate (95% C.I.)	P-value	Covariate Adjusted for MMP9 Status (95% C.I.)	P-value
Lymphatic/Vascular Invasion	67.14 (6.38-2478.80)	< 0.001*	11.92 (1.90-233.48)	0.006*
Tumor Size	16.41 (2.20 -341.79)	< 0.001*	3.19 (1.00-16.94)	0.050*

* Statistically significant at p < 0.05

**Figure 4:**
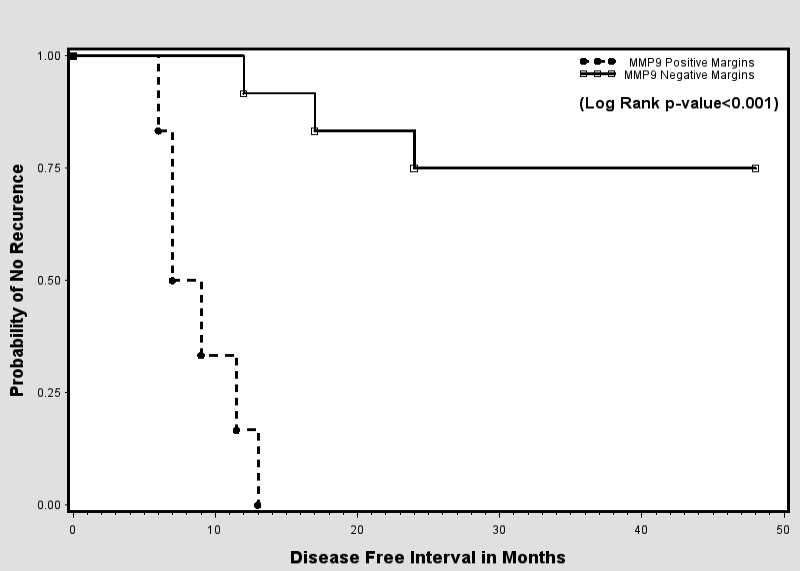
Kaplan-Meier curves showing probability of no recurrence based on MMP-9 status at surgical margins

**Figure 5:**
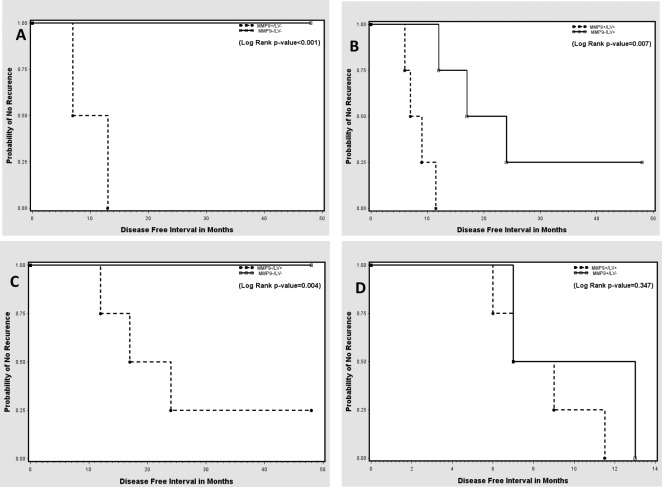
Kaplan-Meier curves showing probability of no recurrence based on combinations of positive- and negative-MMP-9 margins with lymphatic/vascular invasion (LVI) status (A, B) MMP-9 positive and MMP-9 negative margin patients without and with LVI, respectively. (C, D) LVI-positive and LVI-negative patients with MMP-9 negative and MMP-9 positive margins, respectively.

The Kaplan-Meier analysis of the RFT indicated a significant decrease in recurrence-free survival among those with MMP-9-positive margins when compared with those with MMP-9 negative margins (Figure 4). A subset analysis indicated that MMP-9 positivity in the surgical margins yielded a significant decrease in recurrence-free survival regardless of the presence or absence of lymphatic/vascular invasion (LVI) (Figures 5A, 5B). Conversely, with MMP-9-negative margins, there was a significant decrease in recurrence-free survival between LVI-positive and LVI-negative patients (Figure 5C). However, with MMP-9-positive margins, there was no significant difference in recurrence-free survival between the LVI-positive and LVI-negative subgroups (Figure 5D). This demonstrates that LVI had an independent effect on recurrence-free survival when the margins were MMP-9-negative, but not when the margins were MMP-9-positive.

## DISCUSSION

Results of this study indicates that ~45% of histologically negative surgical resection margins of OSCCs express at least one of the oral cancer-associated SIBLINGs and/or one of the MMPs. However, only the expression of DSPP, OPN, and MMP-9 exhibited significant association with prognostic parameters such as recurrence or recurrence-free survival. More significantly, of the SIBLINGS and MMPs investigated, only MMP-9 had an independently predictive association with recurrence-free survival. Although lymphatic/vascular invasion (LVI) and tumor size (T) were also independently predictive when included with MMP-9 in multivariate analyses, their predictive association was not as strong as that of MMP-9. When one considers sensitivity, specificity, and predictive value, dichotomized MMP-9 had the greatest overall accuracy, comparable sensitivity, and greater specificity than the SIBLINGs or the other MMPs when predicting recurrence. MMP-9 was superior in terms of positive predictive value (PV+) and comparable in terms of negative predictive value (PV-). As shown in Table 8, the presence of DSPP and OPN were significantly associated with the presence of MMP-9 such that MMP-9 was not found in the margin of tumors in which both DSPP and OPN were absent. However, neither DSPP nor OPN was independently predictive of recurrence-free survival after accounting for the effect of MMP-9.

To the best of our knowledge, this is the first study investigating the potential significance of the expression of the oral cancer-associated SIBLINGs with their cognate MMPs at histologically-negative surgical resection margins of primary OSCC. Our results indicate that the expression of MMP-9 at histologically-negative surgical resection margins is the preferred predictor of recurrence of OSCC, although it was always co-expressed with DSPP and/or OPN.

The prevailing management philosophy that advocates complete surgical excision with “adequate” tumor-free margins for primary solid tumors such as OSCCs presumes the homogeneity and rectilinear progression of such tumors at the advancing edge [17]. The empirical assumption is that malignancy stopped at visibly defined borders and the surgeon had to cut along the ‘dots’, whilst leaving an adequate margin for error [17; see Figures 1 and 2 schematics]. High recurrence rates for primary OSCCs following excision with histologically negative margins, however, clearly indicates that histologically-negative resection margins about half of the time do not translate into RFS [17-19]. The uncertainty of the precision of resection margins in the surgical treatment of OSCCs is informed by two closely related concepts. First, a single transformed progenitor cell may populate a contiguous area of otherwise normal tissue resulting in multiple, but clonally related foci of potential tumor cells [20]. Alternatively, lateral intraepithelial seeding of a preneoplastic cell into contiguous normal tissues may occur [20]. In both instances, the genetically transformed cell may not have fully assumed phenotypic morphologic characteristics that are apparent by conventional histopathologic parameters. This raises the question as to whether this concept of genetically transformed cells represent the forerunners of the so-called epithelial mesenchymal transition (EMT), a conserved developmental process in which epithelial cells loss E-cadherin-mediated junctions and apical base polarity to become motile and invasive (21,22). Interestingly, MMP-9 (as well as MMP-2) and other metalloproteases are upregulated in the complex process associated with the EMT process, which also involves the intermediate process of the formation of invadopodia (21). Specifically, secreted MMP-9 and MMP-2 have been reported to localize to invadopodia (21).

The complexity in understanding the biology of tumor recurrence at histologically negative resection margins of primary OSCCs is further compounded by the concept of “field cancerization” well documented in human OSCCs [23,24]. Field cancerization presumes occasional multifocal development of oral cancers within the oral cavity such that each foci, although proximate or even contiguous, to adjacent foci may have resulted from different genetic alteration and therefore be clonally different [23,24]. Indeed, studies illustrating the dynamic nature of the field effects of oral cancer resulting from different genetic alterations in different biopsies within a field have been reported [20, 23]. For example, results of a recent study reported by Tsui et al. [19] demonstrated that two genetically unrelated OSCCs may develop within 10mm of each other. Our awareness of this informed our decision to sample at least 10mm of all histologically-negative margins for each case in the present study.

Although recent studies have reported molecular signatures at conventional histologically negative surgical resection margins predictive of the recurrence of OSCC at primary sites, their utility is hampered by their limited practical clinical applications. For example, elf4E and p53 expression at surgical resection margins of primary OSCCs have been suggested as being of predictive value for recurrence at histologically negative resection margins [6, 8]. In the study by Ball et al. [8] paraffin-embedded tissue blocks of surgical margins from 24 patients with OSCCs were immunohistochemically evaluated for the expression of the p53 protein. Fifty-eight percent of the patients had at least one margin positive for p53, including eight of ten patients whose OSCC recurred locally. The sample odds ratio test predicted a 5.333 times higher odds of local recurrence with at least one p53 positive surgical margin. However, the authors did not make it clear whether or not all examined margins were also histologically negative [8].

The studies reported by Nathan et al. [6] analyzed by immnuhistochemistry the expression of elf4E at surgical margins and primary tumors of newly diagnosed head and neck squamous cell carcinoma (HNSCC) patients treated by surgical resection. Their results indicated that all 65 patients had elevated levels of eIF4E in the tumors. Of these, 36 patients (55%) had elevated eIF4E in histologically tumor-free margins out of which 20 (56%) had local-regional recurrences. Of the 29 patients (45%) without eIF4E expression at resection margins, only two of these patients (6.9%) had recurrences [6]. Furthermore, Cox regression analysis indicated that elevated eIF4E in the margins was an independent prognostic factor (P≤ .009) for recurrence, and the Kaplan-Meier curves for the probability of non-recurrence were significantly different for positive and negative eIF4E margins (P≤0001, log-rank test) [6]. The authors therefore concluded that histologically tumor-free surgical margins, expressing eIF4E were predictive of significantly increased risk of recurrence [6]. However, the number of cases in the above study designated as “oral cavity” was 11, and these were without further site-specific analysis of the results [6].

Our current results cast DSPP, OPN, and MMP-9 expression at histologically negative resection margins as potential predictors of recurrence at primary OSCC resection sites, suggesting that true tumor-free margins consistent with RFS may be redefined as histologically-negative-DSPP-OPN-MMP-9-negative resection margins. Multicenter prospective cohort designs are however required to further assess the utility and clinical applicability of the expressions of DSPP, OPN and MMP-9, singly or in combination with DSPP in the overall estimation of tumor-free resection margins consistent with RFS in the surgical management of primary OSCC. Such prospective studies will compare long-term recurrence status of patients with histologically-negative (H-N) surgical resection margins with that of histologically-negative-and DSPP-OPN-MMP-9 negative margins of primary OSCC.

## METHODS

A retrospective study was carried out on archived paraffin surgical resection specimens obtained from patients who underwent surgical resection of their primary OSCC with a curative intent. Only resections with histologically-negative margins were selected for this study. The cases are from patients seen and treated in the Department of Otolaryngology/Head and Neck Surgery at the Georgia Health Sciences University (GHSU) between January 2004 and December 2007. Only cases with adequate follow-up records of at least 4 years were selected for this study. Prior to commencement of study, the required Institutional Review Board (IRB) approval was obtained.

Twenty consecutive cases meeting the inclusion criteria were selected using the archived, initial diagnostic hematoxylin and eosin (H&E) sections. These were reviewed independently by two board-certified Oral and Maxillofacial Pathologists in order to verify the original diagnosis. All the paraffin blocks relating to each case were retrieved and matched with corresponding H&E stained sections. Five micron sections of the histologically negative resection margins were made for immunohistochemistry and analyses for the expression of BSP, DSPP, OPN, MMP-2, MMP-3, and MMP-9 following the steps schematically illustrated in Figures 1 and 2.

### Immunohistochemistry

Antibodies for the SIBLINGs and MMPs used were produced in the laboratory of one of the authors (LWF) and have been previously published [12, 15, 16]. The SIBLING monoclonal antibodies used were LFMb-25 for BSP, LFMb-14 for OPN, and LFMb-21 for DSPP. Their polyclonal counterparts LF-84 (BSP, affinity purified), LF-123 (OPN), and LF-151 (DSPP), respectively, were used to validate corresponding monoclonal antibody results. The Human MMP-2, MMP-3 and MMP-9 were detected using rabbit antibodies generated against MMP-specific synthetic peptides conjugated to keyhole limpet hemocyanin protein through the cysteine in each peptide. (MMP-2, LF-183: ENQSLKSVKFGSIKSDWLGC; MMP-3, LF-182: EPGFPKQIAEDFPGIDSKIDAC; and MMP-9, LF-184: RSELNQVDQVGYVTYDILQCPED) [12]. The antibody was affinity purified in each case using the same peptides conjugated to activated agarose beads. There was no cross-reactivity between each antiserum and the other two authentic human MMPs on ELISA assay. Dilutions of 1:100 (antibody: 10% normal goat serum in PBS) was used for the SIBLING monoclonal antibodies and 1:200 for their polyclonal counterparts [12, 15, 16].

Immunostaining was carried out to localize the SIBLINGs and MMPs on sections using the Zymed ST5050 automated system (Zymed Lab Inc., San Francisco, CA) as previously described [12, 15, 16]. Briefly, 5 μm paraffin sections were manually dewaxed in three xylene washes (5 min each) before rehydrating through graded ethanol (100%, 95%, and 75%) and deionized water. Endogenous peroxidase activity was destroyed by treating sections for 30 min with 3% hydrogen peroxide in methanol. Sections were thereafter washed 3 times in phosphate-buffered saline (PBS) for 5 min each and covered with PBS + 0.05% Tween-20 (PBS-T) before loading the slides onto a preprogrammed ST5050 automated immunohistochemistry machine. Sections were incubated for 1 h with appropriate SIBLING/MMP antibody diluted in 10% normal goat serum in PBS. The sections then underwent a 4×1 min wash cycle with PBS-T and incubated with SuperPicTure Polymer horseradish-peroxidase (HRP)-conjugated broad-spectrum secondary antibody (#87-8963, Zymed Lab. Inc., San Fransisco, CA, USA) for 10 min. Thereafter, sections were washed in PBS before developing with AEC (amino ethyl carbazol) Single Solution chromogen (#00-1122, Zymed Lab. Inc., San Francisco, CA, USA) for 2 min. Counterstain with Mayer's hematoxylin for 10 sec was carried out manually before applying an overlay of Clearmount (Zymed Lab. Inc., San Francisco, CA) glaze. After drying, slides were coverslipped with Histomount (Zymed Lab. Inc., San Francisco, CA, USA). All steps were performed at room temperature. For negative controls, primary antibodies were substituted with either non-immune rabbit serum or mouse IgG control (#08-6599, Zymed Lab. Inc., San Frencisco, CA, USA). Photographic images of representative reproducible results were captured using the Axioplan 2 Universal microscope equipped with an Axiovision digital camera and Axiovision program (Carl Zeiss Gmbh, Jena, Germany).

### Scoring of Immunohistochemistry results

Immunoreactivity to each SIBLING and MMP was scored semiquantitatively by two independent pathologists who were blinded to the clinicopathological details of all cases until after the completion of the scoring. A co-investigator (PW) not involved with the immunohistochemistry scoring retained the clinical data (summarized in Table 1) of the patients for all selected cases, while the co-investigators involved with the experiments and scoring were blinded from the clinical and follow-up information until after the study was completed. Initial scoring was as follows: 0 (not detectable or <10% of immunoreactive tumor cells); 1+ (>10% but <50% of immunoreactive tumor cells); 2+ (>50% but <75% of immunoreactive tumor cells); and 3+ (widely and highly expressed in tumor cells). Any extra-epithelial staining was also documented.

### Statistical Methods

All statistical analyses were performed using SAS software (Version 9.2, SAS Institute, Inc., Cary, N.C., 2008). For statistical analysis, each scoring set for SIBLING and MMP was dichotomized as “present” (≥10%) or “absent” (<10%). The Sensitivity, Specificity, Positive Predictive Value (PV+) and Negative Predictive Value (PV-) for recurrence of OSCC were calculated for each dichotomized SIBLING and MMP. The overall diagnostic accuracy for each SIBLING and MMP was calculated as (number of true positives + number of true negatives) / number of patients. The exact binomial test with mid-p correction was used to determine any significant difference between OSCC recurrence rate observed on the present sample and the estimated population recurrence rate of 50%. Fisher's exact test (two categories) and the Fisher-Freeman-Halton test (more than two categories) were used to examine the associations between clinical variables (summarized in Table 1), and recurrence rate as well as any association between SIBLING or MMP expression at the surgical margin and each patient's characteristics/clinical variables. The Kaplan-Meier method and the log-rank test were used to examine the association of each SIBLING or MMP expression with recurrence-free survival. Multivariate Cox regression with forward selection was performed for each combination of SIBLING (or MMP) and each of the clinical factors significantly associated with recurrence-free survival at the 0.05 level in the univariate Cox regression analyses. These analyses were performed to determine any independent effects of the SIBLING or MMP (controlling for significant covariates) on recurrence-free survival. Continuous variables were summarized using mean ± standard deviation (S.D.).
